# A multi-centre, parallel group superiority trial of silk therapeutic clothing compared to standard care for the management of eczema in children (CLOTHES Trial): study protocol for a randomised controlled trial

**DOI:** 10.1186/s13063-015-0921-9

**Published:** 2015-09-02

**Authors:** Eleanor F. Harrison, Rachel H. Haines, Fiona Cowdell, Tracey H. Sach, Taraneh Dean, Ian Pollock, Nigel P. Burrows, Hannah Buckley, Jonathan Batchelor, Hywel C. Williams, Sandra Lawton, Sara J. Brown, Lucy E. Bradshaw, Amina Ahmed, Alan A. Montgomery, Eleanor J. Mitchell, Kim S. Thomas

**Affiliations:** Nottingham Clinical Trials Unit, University of Nottingham, Nottingham Health Science Partners, Queen’s Medical Centre, Nottingham, NG7 2UH UK; Faculty of Health and Social Care, University of Hull, Room 204, Dearne Building, Hull, HU6 7RX UK; Norwich Medical School, University of East Anglia, Norwich Research Park, Norwich, NR4 7TJ UK; School of Health Sciences and Social Work, University of Portsmouth, James Watson Building, 2 King Richard Road, Portsmouth, PO1 2FR UK; Royal Free London NHS Foundation Trust, Barnet Hospital, Wellhouse Lane, Barnet, Hertfordshire, EN5 3DJ UK; Department of Dermatology, Cambridge University Hospitals NHS Foundation Trust, Addenbrooke’s Hospital, Hills Road, Cambridge, CB2 0QQ UK; Portsmouth Hospitals NHS Trust, Queen Alexandra Hospital, Southwick Hill Road, Cosham, PO6 3LY UK; Centre of Evidence Based Dermatology, University of Nottingham, Nottingham, NG7 2UH UK; Nottingham University Hospitals NHS Trust, Queens Medical Centre, Nottingham, NG7 2UH UK; Dermatology and Genetic Medicine, Division of Cancer Research (JWCC level 7), Medical Research Institute, University of Dundee, Ninewells Hospital, Dundee, DD1 9SY UK; Patient and Public Involvement representative, Nottingham, UK

**Keywords:** Eczema, Silk, Clothing, Fabric, Atopic eczema, Atopic dermatitis, RCT

## Abstract

**Background:**

Eczema is a chronic, itchy skin condition that can have a large impact on the quality of life of patients and their families. People with eczema are often keen to try out non-pharmacological therapies like silk therapeutic garments that could reduce itching or the damage caused by scratching. However, the effectiveness and cost-effectiveness of these garments in the management of eczema has yet to be proven. The CLOTHES Trial will test the hypothesis that ‘silk therapeutic garments plus standard eczema care’ is superior to ‘standard care alone’ for children with moderate to severe eczema.

**Methods/Design:**

Parallel group, observer-blind, pragmatic, multi-centre randomised controlled trial of 6 months’ duration.

Three hundred children aged 1 to 15 years with moderate to severe eczema will be randomised (1:1) to receive silk therapeutic garments plus standard eczema care, or standard eczema care alone. Primary outcome is eczema severity, as assessed by trained and blinded investigators at 2, 4 and 6 months (using the Eczema Area and Severity Index (EASI)). Secondary outcomes include: patient-reported eczema symptoms (collected weekly for 6 months to capture long-term control); global assessment of severity; quality of life of the child, family and main carer; use of standard eczema treatments (emollients, corticosteroids applied topically, calcineurin inhibitors applied topically and wet wraps); frequency of infections; and cost-effectiveness. The acceptability and durability of the clothing will also be assessed, as will adherence to wearing the garments. A nested qualitative study will assess the views of a subset of children wearing the garments and their parents, and those of healthcare providers and commissioners.

Randomisation uses a computer-generated sequence of permuted blocks of randomly varying size, stratified by recruiting hospital and child’s age (< 2 years; 2 to 5 years; > 5 years), and concealed using a secure web-based system. The sequence of treatment allocations will remain concealed until randomisation and data collection are complete.

Recruitment is taking place from November 2013 to May 2015, and the trial will be completed in 2016. Full details of results will be published in the National Institute for Health Research Journal series.

**Trial registration:**

Current Controlled Trials ISRCTN77261365 (registered 11 November 2013).

## Background

Eczema (synonymously atopic eczema, atopic dermatitis) is a chronic, inflammatory skin condition that affects around one in five children and appears to be increasing in prevalence worldwide [1]. Whilst most cases of eczema can be successfully treated with topical medications, many parents express inconvenience and/or concern in using these preparations and are keen to explore non-pharmacological treatment options.

Some types of clothing can cause irritation to the skin, and current guidelines recommend the use of loose cotton clothing, and the avoidance of wool and other rough fibres next to the skin [2]. In response to this need, new clothing products have become available in recent years, with many marketed as having beneficial effects in the treatment of eczema. The therapeutic silk garments included in this study are available on prescription in the UK, at a cost ranging from £35.15 to £52.54 for a child’s bodysuit/top and £24.95 to £32.49 for child’s leggings [3]. However, the evidence from randomised controlled trials (RCTs) supporting the use of these garments is currently limited [4, 5].

Searching the Global Resource of Eczema Trials [6] (last search date: 19 March 2015), 9 small RCTs assessing the effects of therapeutic clothing have been published to date. Two trials investigated silver-coated textiles [7, 8]; one investigated cellulose fibres with seaweed enriched with silver ions [9]; one investigated an anion textile [10]; two investigated types of ethylene vinyl alcohol fibre [11, 12] and three investigated silk clothing (DermaSilk™, Espère Healthcare Ltd, Shefford, UK) [13–15].

The 3 silk clothing trials were too small (22, 30 and 22 participants respectively) to provide robust evidence and inform clinical practice. Due to the limited evidence available, the National Institute for Health Research Health Technology Assessment Programme (NIHR HTA) prioritised the need for a large pragmatic trial to establish whether or not silk therapeutic clothing is cost-effective in the management of children with eczema. This led to a funding call in 2011 with the subsequent commissioning of the CLOTHing for the relief of Eczema Symptoms Trial (CLOTHES ID: 11/65/01). The chosen comparator group was standard eczema care, so that the additional benefits of silk therapeutic clothing as an adjuvant to standard care could be assessed.

The CLOTHES Trial has been developed with support from the UK Dermatology Clinical Trials Network, and delivered with assistance from the National Institute for Health Research Comprehensive Local Research Network.

## Objectives

Research hypothesis: that silk therapeutic clothing plus standard eczema care is superior to standard eczema care alone.

The primary objective is to assess whether silk therapeutic clothing, when used in addition to standard eczema care, reduces eczema severity in children with moderate to severe eczema over a period of 6 months.

Secondary objectives are to i) estimate the ‘within trial’ cost-effectiveness of silk therapeutic clothing from a National Health Service (NHS), and separately a family, perspective; ii) to explore parent/guardian and child views on, and experiences of, using silk garments and factors that might influence the use of these garments in everyday life; and iii) to examine prescribers’/commissioners’ views of the use of silk garments, to inform prescribing practice.

## Methods/Design

The CLOTHES Trial is a multi-centre, parallel group, observer-blind, pragmatic RCT aiming to recruit 300 children with moderate to severe eczema. Participants are randomised to 2 groups using an allocation ratio of 1:1, and are followed up for a period of 6 months. The primary outcome is eczema severity measured using a validated scale by trained and blinded research nurses at baseline and 2, 4 and 6 months after randomisation.

Participants randomised to therapeutic clothing are further randomised (1:1) to receive one of the two brands of clothing used in the trial (DermaSilk™, Espère Healthcare Ltd, Shefford, UK or DreamSkin™, DreamSkin Health Ltd, Hatfield, UK).

At the end of the 6-month RCT, participants in the standard care control arm are provided with silk garments so they have the opportunity to try the garments for themselves, and to encourage retention in the trial. All participants are followed up for a further 2 months (observational period).

During the first 6 months of trial recruitment, an internal pilot was conducted to assess ability to recruit, adherence with the intervention, and retention in the trial. The criteria for continuation (≥ 50 % recruitment and retention) were met and the main trial has continued as planned. Adherence with the trial intervention, quantified as wearing the trial garments more than 50 % of the time, was also very high amongst trial participants (87.5 %) over this period.

Alongside the trial, qualitative methods are being used to explore barriers and facilitators to the use of the silk garments amongst participants in the trial (children and their parent/guardian), healthcare professional prescribers, and healthcare commissioners. Participants/parents are invited to take part in interviews/focus groups once their participation in the main trial has ended (8 months). Prescribers and commissioners have been identified via existing professional networks, and structured telephone interviews conducted.

**Fig. 1:**
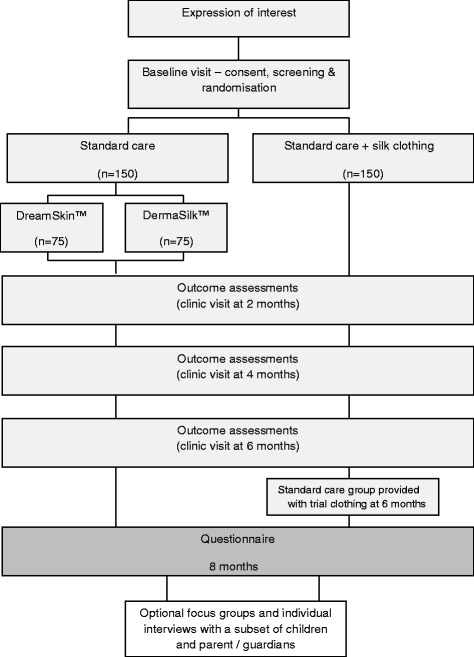
Trial flowchart

Flow of participants through the trial is summarised in Fig. 1.

### Participants, interventions and outcomes

#### Setting

Recruitment is taking place in five recruiting centres in the United Kingdom: Queen’s Medical Centre, Nottingham University Hospitals NHS Trust; Chase Farm Hospital, Royal Free London NHS Foundation Trust; Addenbrooke’s Hospital, Cambridge University Hospitals NHS Foundation Trust; Queen Alexandra Hospital, Portsmouth Hospitals NHS Trust and St Mary’s Hospital, Isle of Wight NHS Trust. Sites were chosen on the basis of a proven track record of recruiting into previous eczema trials.

Potential participants are identified through secondary care, primary care and through local advertising (self-referral).

#### Eligibility

##### Inclusion criteria

- Aged 1 to 15 years at baseline
- Diagnosis of moderate or severe eczema. Presence of eczema is confirmed using the UK Diagnostic Criteria for Atopic Eczema [16] and eczema severity judged using the Nottingham Eczema Severity Scale (NESS) [17]
- Resident within travelling distance of a recruiting centre
- At least one patch of eczema on the trunk or limbs
- Parent/legal guardian able to give informed consent and child willing to take part in the trial

##### Exclusion criteria

- Taken systemic medication (including light therapy) or steroids taken orally for eczema within the previous 3 months
- Started a new treatment regimen within the last month
- Used wet/dry wraps ≥ five times in the last month
- Currently using silk clothing for their eczema and are unwilling to stop using the clothing during the trial
- Currently taking part in another clinical trial

Only one child will be enrolled per family.

The decision was taken to exclude children under 1 year as it is often difficult to diagnose atopic eczema in very young children (as distinct from irritant dermatitis or seborrheic dermatitis). Also in this age group children are growing rapidly making it likely that they would require replacement garments within the 6-month trial period.

#### Interventions

Participants are randomised to receive either standard eczema care plus silk garments, or standard care alone.

##### Silk garments

The medical device under investigation is a knitted, sericin-free silk therapeutic garment with a CE mark for use in eczema. Sericin is a protein that coats the outside of silk fibres and has the potential to cause allergic reactions. Medical-grade silk (such as silk used for stitches during operations) has the sericin removed for this reason.

The two products being used are DermaSilk™ (Espère Healthcare Ltd, Shefford, UK) and DreamSkin™ (DreamSkin Health Ltd, Hatfield, UK), as these were the two brands of silk garments available on prescription at the time of trial design. Participants are instructed to wear the clothing as often as possible during the day and at night. Participants receive three sets of garments (long-sleeved vest and leggings, or body suits and leggings depending on the age of the child). This was thought to be a sufficient number of garments to allow for continual use between washes. Washing instructions are provided in line with manufacturers’ recommendations.

The product is prepared at the trial coordinating centre by removing branding labels and packed into blinded, trial-specific packaging. Upon randomisation, garments are sent directly to participants’ homes from the coordinating centre, to maintain the blinding of local site staff. Garments are replaced as required during the 6-month RCT (e.g. because they are worn out or because the child has grown). Once the randomised part of the trial is complete at 6 months, garments are not replaced.

##### Standard care

All participants continue with their standard eczema care in line with National Institute for Health and Care Excellence (NICE) guidance [18] including emollients, corticosteroids applied topically and calcineurin inhibitors applied topically. No efforts are made to intervene in or change a child’s standard eczema care unless the research nurse thinks that the skin may be infected. If a research nurse suspects infection they recommend that the participant contacts their normal medical team for confirmation of infection and subsequent treatment or change to eczema management.

If a child is currently using ‘specialist’ cotton clothing (e.g. special sleep suits with built-in mittens), the use of these garments is recorded, but is not grounds for exclusion.

##### Adherence

Adherence to wearing the garments is collected on weekly questionnaires until 6 months and then again at the end of the study (8 months). Participants randomised to standard care plus silk garments have the option of using a sticker or tick chart to record the days and nights when they wear the clothing for the first 6 months. This acts as an aide-mémoire for questionnaire completion.

##### Concomitant medication

All participants continue with their standard eczema care; however, they are asked not to start any new treatments for their eczema (other than antibiotics for skin infection) during the period of the trial. Participants are asked to refrain from using other prescription clothing and discouraged from routinely using bandages or wet or dry wrap dressings. Any change to a child’s treatment regimen, or use of wet/dry wrapping, is recorded but the child remains in the trial.

#### Outcomes

Core outcome domains for collection in eczema trials have been defined as being eczema signs, eczema symptoms, quality of life and long-term control of flares [19]. All four of these outcome domains are included in the CLOTHES Trial.

##### Primary outcome

The primary outcome is eczema severity measured by the objective Eczema Area and Severity Index (EASI) [20] assessed by blinded research nurses at baseline and 2, 4 and 6 months after randomisation.

EASI was chosen as the primary outcome as it is a validated scale that has been recommended as part of the core outcome set for use in eczema trials [21]. The scale captures eczema signs (erythema (redness), excoriation (scratching), oedema/papulation (swelling and fluid in the skin) and lichenification (thickening of the skin)). It is suitable for use in capturing eczema severity by independent observers who are blinded to treatment allocation.

##### Secondary outcomes

- Self-reported eczema symptoms, assessed weekly using the Patient Oriented Eczema measure (POEM) [22]. By capturing self-reported eczema severity every week for the duration of the trial (6-month RCT), we will capture long-term control of flares as well as self-reported eczema symptoms. The POEM is also collected, once, at the end of the observational period (8 months).
- Global assessment of the eczema, assessed by research nurses (Investigator Global Assessment: IGA) and by participants (Participant Global Assessment: PGA) at baseline, 2, 4 and 6 months. This outcome is included to aid interpretation of the trial findings.
- Three Item Severity scale (TIS) [23] at baseline, 2, 4 and 6 months, assessed by the research nurses and used to assess eczema severity. Given the importance of an objective measure to capture eczema severity in this observer-blind trial, it was felt that a second validated eczema severity scale was warranted.
- Use of eczema treatments: days of use of steroids applied topically, calcineurin inhibitors applied topically, emollients and wet/dry wrapping throughout the trial. This outcome is important for helping to interpret the trial results if use of the silk garments results in changes in the frequency of application of standard eczema treatments (or changes in potency).
- Number of skin infections – defined as patient-reported skin infections that required antibiotic or antiviral treatment. Silk therapeutic garments are purported to reduce the incidence of clinically-relevant skin infections, but it is also possible that long-term use of the garments could exacerbate skin infections. This is an important safety outcome for the trial.
- Health-related quality of life at baseline and at 6 months: Dermatitis Family Impact (DFI) [24] will assess the impact of the child’s health on the whole family unit, EuroQol Five Dimension Three Levels (EQ-5D-3 L) will provide a utility measure for the main carer; Atopic Dermatitis Quality of Life preference-based index (ADQoL) [25] and Child Health Utility, nine dimensions (CHU-9D) [26] will provide eczema-specific and general health utility scores respectively.
- Health resource use, along with f) health-related quality of life, will inform the within trial cost-effectiveness analysis.

Durability of the garments, adherence and acceptability of use (as assessed by children and parents/carers) is also assessed.

##### Tertiary outcomes

Additional exploratory analysis will be conducted based on eczema severity scores in areas covered by the clothing (body and limbs) compared to areas uncovered by the clothing (head and neck), in order to test the theory that gaining eczema control in one site may reduce a patient’s overall immunological response and, therefore, disease activity at distant sites.

Whilst it is assumed that the different brands of clothing are similar, the effects of receiving different brands of clothing will be explored.

All tertiary outcomes will be considered exploratory.

#### Participant timeline

Recruitment will take place from November 2013 to May 2015. Each participant is enrolled in the study for 8 months in total (6-month RCT, followed by a 2-month observational period).

Details of the data collection schedule are summarised (Table 1).

**Table 1:** Timetable of study assessments

	RCT				Observational period
Outcomes collected	0 months	2 months	4 months	6 months	8 months
Informed consent for main study	√				
Informed consent for genetic study (optional)	√				
Eligibility checks	√				
Eczema severity (NESS)	√				
Supply garments	√ intervention			√ control	
Demographics	√				
Collect saliva sample (optional)	√				
Issue diary	√	√	√	√	
EASI and TIS	√	√	√	√	
Investigator and Patient Global Assessment (IGA and PGA)	√	√	√	√	
Topical treatment usage	√	√		√	
Medication for skin infection	√	√	√	√	
Use of wet and dry wraps	√	√	√	√	
On-line questionnaire (weekly) including POEM, topical treatment use and use of wet and dry wraps		√	√	√	
Final on-line questionnaire					√
DFI	√			√	
EQ-5D-3 L of parent	√			√	
Child utility scales (ADQoL, CHU-9D)	√			√	
Number of infections	√	√	√	√	
Serious adverse events		√	√	√	
NHS and family resource use	√	√ & Diary	√ & Diary	√ & Diary	√
Adherence		Ques	Ques	Ques	Ques
Durability of clothing				√	√
Acceptability (parent and child)				√	√
Replace garments if required (intervention group only)		√	√		

*CHU-9D –* The Child Health Utility, nine dimensions, *DFI* Dermatitis Family Impact questionnaire, *EASI* Eczema Area and Severity Index, *EQ-5D-3 L* – EuroQol Five Dimension, Three Levels questionnaire, *NESS* Nottingham Eczema Severity Scale, *POEM* Patient Oriented Eczema Measure, *Ques* = weekly on-line/postal questionnaire, *TIS* Three Item Severity scale

At 8 months, parents/guardians are sent an invite to the qualitative component of the study, as well as parent and age-appropriate child information sheets. Patients and their families may choose to contact the research team to express interest in participating in the interviews/focus groups.

#### Sample size

Three hundred participants provides 90 % power, at the 5 % significance level (2-tailed) to detect a difference of around 3 points between the groups in mean EASI scores over 2, 4 and 6 months using a repeated measures analysis of covariance (ANCOVA), assuming a SD of 13, a correlation between EASI scores at different time points of 0.6 and loss to follow-up of 10 %.

Although a 3-point improvement in EASI sounds like a small change, it represents a clinically meaningful difference between groups. A small treatment response could still be worthwhile to the NHS since this non-pharmacological treatment is assumed to have no adverse effects, and eczema is widely prevalent among the general population. It is also likely that a relatively small response on the objective primary outcome will be reflected in larger, more clinically meaningful treatment effects in the patient-reported outcomes.

#### Recruitment

Recruitment is based in five secondary care hospitals. Recruitment has primarily been from dermatology and paediatric allergy clinics at the local sites and through self-referral as the result of direct local advertising (articles in press, radio and TV interviews, websites and forums). General practice (GP) surgeries and other local hospitals in the surrounding area are also being used as Patient Identification Centres (PICS).

If needed, the usual hospital interpreter and translator services is available to assist with discussion of the trial, but the consent forms and information sheets are not available in other languages.

### Assignment of interventions

#### Randomisation and blinding

The randomisation schedule is based on a computer-generated pseudo-random code using random permuted blocks of randomly varying size, created by the Nottingham Clinical Trials Unit (Nottingham CTU) in accordance with their standard operating procedure (SOP) and held on a secure University of Nottingham server. Randomisation is stratified by recruiting hospital and by child’s age: < 2 years; 2 to 5 years; and > 5 years.

Investigators and research nurses access the randomisation website by means of a remote, Internet-based randomisation system developed and maintained by the Nottingham CTU. The sequence of treatment allocations will be concealed until interventions have all been assigned and recruitment, data collection, and all other trial-related assessments are complete.

After each allocation, the Nottingham CTU coordinating centre is notified so that participants can be informed by letter of their treatment allocation, and receive their supply of therapeutic clothing if appropriate. Upon entry into the trial, participants in the therapeutic clothing arm are further randomly allocated to one of two therapeutic clothing brands available on prescription in the UK. They are not aware which brand of clothing they have received. Packaging, labelling and distribution of clothing is performed by staff at the coordinating centre and sent to participants by post. Sizes are determined by collection of the participant’s height at randomisation

Whilst it is not possible to blind participants to their treatment allocation, efforts have been made to minimise expectation bias by emphasising in the trial documents that the evidence supporting the use of therapeutic clothing for eczema is currently limited, and that we do not yet know if this clothing offers any benefit over standard care. The participant-facing study documents also avoid the use of value-laden terms such as ‘specialist’ or ‘therapeutic’ clothing for the same reason.

Where possible, research nurses remain blinded throughout the trial: participants are reminded in the study literature and in their clinic appointment letters not to wear the clothing when they attend the clinic, or to mention the clothing in any way when talking to the research nurses. All questions relating to the acceptability and use of the clothing are completed by either postal or on-line questionnaire (according to patient preference), and telephone and Email contact with participants is made by trial staff from the coordinating centre whenever possible. If the research nurses become unblinded, this is recorded and will be used to inform a sensitivity analysis.

Full details of blinding arrangements are summarised (Table 2).

**Table 2:** Summary of blinding status

	Blinding status	Comments
Participants	Not blinded	Not possible to blind participants, efforts will be made to minimise expectation bias
Research nurses and PI	Blinded	Participants will be reminded in their clinic appointment letters not to wear the clothing when they attend the clinic, or to mention the clothing in any way when talking to the research nurses
Trial staff at Nottingham CTU	Not blinded	Will be the main point of contact for participants wishing to contact the research team, will package and post the clothing to the participants according to the randomisation schedule, and will provide general advice
Statistician	Blinded	Statistician will finalise the analysis plan prior to revealing the treatment codes

*CTU* Clinical Trials Unit, *PI* Principal Investigator

### Data collection, management and analysis

#### Data collection and methods to ensure quality and retention

Data collection and skin assessments are made by research nurses who have been trained in using the EASI and TIS instruments, and who are blinded to group allocation. The same research nurse assesses the skin at all time-points for each participant in order to minimise inter-observer variability.

On-line questionnaires (or paper questionnaires if preferred) are used to collect self-reported weekly outcomes, with Email reminders sent on the day the questionnaire is due to be completed, and again 2 days later if it has not been completed. Where participants/parents fail to complete the questionnaire at 6 and 8 months, or do not complete weekly questionnaires for 3 consecutive weeks, attempts are made by the coordinating centre to contact the family for follow-up. This is done via telephone in the first instance, and followed up with an Email if necessary. If a response is not received within a week, a paper copy of the questionnaire is posted to the parent with a reply paid envelope.

Participants who fail to attend clinic visits at 2 and/or 4 months continue to be invited to subsequent follow-up visits unless they opt to withdraw from trial follow-up.

Data collection and retention rates are being monitored by the Trial Management Group (TMG) throughout the trial.

Data from the qualitative study will be linked with data from the RCT to provide a rounded assessment of the impact of wearing silk garments and the patient-reported factors that may influence this.

Data collection tools are available at www.nottingham.ac.uk/CLOTHES.

#### Data management

Clinic data are entered directly into a web-based trial database at investigator sites by site users with unique login details. Patient Questionnaires completed at clinic visits are transcribed by the site nurses directly into the trial database.

Patient Questionnaires are completed by participants at home, via an on-line system built and maintained by the Nottingham CTU. For the few participants who prefer a paper format, paper questionnaires are returned in a reply paid envelope to the Nottingham CTU for data entry into the trial database.

Data quality is ensured by database validation checks which include missing data, out of range values, illogical entries and invalid responses. Data entered by sites into the trial database are subject to monitoring and review by coordinating centre staff, and data queries are raised as necessary.

Detailed data management processes and procedures are documented in the CLOTHES Data Management Plan.

#### Statistical methods

All analyses will be carried out using Stata/SE 13.1 (StataCorp, College Station, TX, USA), or MLwiN v2.2. Appropriate descriptive statistics will be used to compare the randomised arms at baseline. The primary approach to analysis will be to analyse participants as randomised (intention-to-treat), regardless of adherence with allocation, without imputation of missing outcome data. Estimates of the intervention effect will be presented with 95 % confidence intervals and exact *p* values. Regression models will include the stratification variables site and age.

The primary analysis for the total EASI score will be performed using a multilevel model, with observations at 2, 4 and 6 months (level 1) nested within participants (level 2) and including baseline EASI and the stratification variables as covariates. This model will use all the observed data and makes the assumption that missing EASI scores are missing at random given the observed data. The effect of trial clothing on eczema severity changing over the study period will be investigated by including an interaction term between treatment group and time point in the model. If there is no evidence of a differential effect over time, a single treatment effect will be reported. If there is evidence of an interaction effect then the treatment effect at each different time point will be reported. The assumptions for the multilevel model will be checked. Appropriate transformations will be considered if the assumptions are violated.

Sensitivity analyses for the primary outcome will take into account adherence with allocation and missing outcome data.

Analyses of secondary outcomes will be conducted using appropriate multivariable regression models. Differences in means for the intervention group compared to the standard care group will be presented for continuous outcomes and risk differences and relative risks for binary outcomes.

Adherence with the trial clothing will be summarised using the proportion of days and nights that the study clothing was worn. This will be done for participants where at least half of the weekly questionnaires were completed. Sensitivity analyses will explore adherence for all participants by making different assumptions about clothing wear during periods where the questionnaire was not completed. Durability and acceptability of use of the garments will be summarised descriptively.

Full details of the planned analyses, including the tertiary outcomes, will be documented in the Statistical Analysis Plan which will be finalised prior to release of treatment allocation codes for analysis. Any changes in the planned statistical methods will be documented in the trial report and a copy of the Statistical Analysis Plan will be made available at www.nottingham.ac.uk/CLOTHES [27].

##### Planned sub-group analysis

Saliva samples are collected for DNA extraction to test for mutations on the gene encoding for filaggrin (*FLG*) to inform a planned sub-group analysis based on presence or absence of loss of function mutation(s) in *FLG*.

For the sub-group analysis, study participants will be tested for up to 6 of the most prevalent *FLG* loss-of-function mutations, depending on quality/quantity of DNA (R501X, 2282del4, R2447X, S3247X, 3702delG and 3673delC) and categorised into 2 groups according to *FLG* genotype:

1. *FLG* wild-type (no mutations) – control cohort
2. *FLG* heterozygous (carrying 1 *FLG* null mutation) and *FLG* homozygous or compound heterozygous (carrying 2 *FLG* null mutations)

##### Health economic evaluation

The base case will compare the within trial cost-effectiveness of silk clothing with standard care to standard care alone from an NHS perspective. A family perspective will be presented separately. An incremental cost analysis will compare the mean per patient costs for the intervention to standard care, measuring resource use such as primary care contacts, medication prescribed, and secondary care contacts. Health and family resource use data will be recorded by research nurses. The patients will be asked to collect data on a diary which will serve as an aide-mémoire. Resource use will be valued for a common price year using published unit costs (e.g. Curtis and Netten [28], BNF 2015 [3], and NHS reference costs) and patient reported estimates. The costs to the NHS and patient will be reported separately as well as in combination.

The primary measures of effectiveness for the cost-effectiveness analysis will be the difference in number of participants who achieved ‘treatment success’ (defined as those with at least a 50 % improvement in EASI at 6 months compared to baseline). Secondary analyses will be conducted using continuous data from the EASI scale; the Dermatitis Family Impact Scale (DFI) [24]; eczema specific utility measure: the Atopic Dermatitis Quality of Life preference-based index (ADQoL) [25] and generic measures of health utility as measured using the EQ-5D-3 L (for the main carer) and the CHU-9D [26] for children aged 5 and over (parental proxy for 5 and 6 year olds and self-completing for those aged 7 and over). A cost utility analysis where effectiveness is measured in terms of the Quality Adjusted Life Years (QALYs) for child and main carer will be undertaken (using linear interpolation and area under the curve with baseline adjustment) [29].

If non-dominance occurs an incremental cost-effectiveness ratio will be produced. Decision uncertainty will be illustrated using a cost-effectiveness acceptability curve. Sensitivity analysis will be undertaken to test the robustness of results in the face of any uncertainties or assumptions made in the analysis.

##### Analysis of qualitative component

Data from the parent/guardian and child focus groups and interviews and prescriber and commissioner interviews will be analysed separately. Parent/guardian and child data will be analysed using a manual process of thematic analysis. Audio tapes will be transcribed verbatim. Transcripts, notes and artefacts (as explained by children) will be reviewed in detail, coding the substance of the data. Theoretical codes will be added beside each code as a reminder of any thoughts or questions attached to the codes. As codes develop they will be compared with the aim of identifying similarities and differences amongst incidence in the data. The identified codes will be condensed into higher levels of abstraction to form categories; these will be continuously reviewed to ensure that they cover variations within the data. The final step will be to interpret the categories so that better understanding is achieved. Prescriber and commissioner interviews will be analysed using the five steps of framework analysis.

### Monitoring

#### Data monitoring

Integrity of trial conduct is overseen by a Trial Steering Committee (TSC), which meets at least once a year and provides overall supervision of the trial on behalf of the trial sponsor (University of Nottingham).

The Trial Management Group (TMG) meets more frequently and is responsible for the day-to-day management of the trial. Members of the TMG report to the TSC at their annual meetings.

As adverse events (AEs) related to the clothing are unlikely, there is no Data Monitoring Committee, and independent oversight of trial data collection, management and analysis is undertaken by the TSC.

The chief investigator (CI) has overall responsibility for the study and is custodian of the data.

#### Interim analyses

There are no planned interim between-group analyses. However, progress with recruitment and retention is monitored monthly by the TMG. If progress is below target, strategies will be implemented to improve progress in discussion with the TSC.

#### Assessment of harm

It is unlikely that silk garments will result in any adverse effects. As such collection of AEs, beyond those listed as trial outcomes, is limited to recording serious adverse events (SAEs).

A worsening of eczema and infected eczema are not recorded as AEs as these are collected as specific outcomes for the trial. Hospitalisation due to eczema is captured as a SAE.

### Ethics and dissemination

#### Research ethics approval

Ethics approval was granted by NHS Health Research Authority, NRES Committee East Midlands – Nottingham 1, and the respective NHS Research & Development (R&D) departments for participating sites (Nottingham University Hospitals NHS Trust; Barnet and Chase Farm Hospitals NHS Trust, Cambridge University Hospitals NHS Foundation Trust, Portsmouth Hospitals NHS Trust and Isle of Wight NHS Trust) prior to start of recruitment. The trial is being conducted in accordance with the ethical principles that have their origin in the Declaration of Helsinki, 1996; the principles of Good Clinical Practice, and the Department of Health Research Governance Framework for Health and Social care, 2005.

#### Protocol amendments

The methods described in this protocol reflect the current protocol (v 3.0 dated 11 February 2014). A summary of protocol amendments are summarised (Table 3).

**Table 3:** Summary of protocol amendments that impacted on trial design

Protocol	Date	Summary of changes prior to start of recruitment
V 2.0	1 August 2013	• Eligibility criteria amended to include children with at least one patch of eczema on the trunk or limbs
Protocol	Date	Summary of changes after start of recruitment
V 3.0	11 February 2014	• The number for filaggrin genotype mutations which the lab will be looking for has been increased from 4 to 6, to now also include: ‘up to 6 mutations’ (depending on quantity/quality of DNA) and the additional two are 3702delG and 3673delC
		• Introduction of details of optional qualitative component for study participants (parents and children) to take part in at the end of the 8-month RCT

*RCT* randomised controlled trial

All amendments to the protocol and associated paperwork have been approved by the trial sponsor, research ethics committee, local R&D departments, and trial funder prior to implementation.

### Consent

Age-appropriate participant information sheets are provided for parents/guardians and children, and they have the opportunity to discuss the study before agreeing to take part. Participants are identified and contacted by their normal care provider in the first instance. If they wish to take part they are asked to contact the research team and provide contact details. Contact details are stored securely and separately from other trial data. All other data are anonymised.

The process for obtaining assent and parent/guardian informed consent is in accordance with the research ethics committee guidance, and Good Clinical Practice (GCP). The investigator, or their nominee, and the participant or other legally authorised representative both sign and date the Informed Consent Form before the person can participate in the study. Children are able to provide assent to participation in the trial if they wish to. Separate (optional) consent to provide a saliva sample for genetic testing to inform a planned sub-group analysis is taken.

No trial-specific procedures are conducted before informed consent has been obtained, and participants are reminded that they may withdraw from the trial at any time without it affecting the quality of their care in the future.

#### Confidentiality

Individual participant medical information obtained as a result of this study is confidential. Participant confidentiality will be ensured using identification code numbers to correspond to treatment data in the computer files. Patient identifiable information will be stored in locked filing cabinets in a secure room.

Medical information may be given to the participant’s medical team and all appropriate medical personnel responsible for the participant’s welfare.

#### Access to data

Data generated as a result of this trial will be available for inspection on request by the participating physicians, the University of Nottingham representatives, the research ethics committee, local R&D departments and the regulatory authorities.

Requests for access to the original anonymised dataset should be made to the CI.

#### Post-trial care

After completing the study participants will continue to receive their standard eczema care through the NHS in accordance with local practice. All participants can keep the clothing provided during the trial, to wear as little or as often as they choose. A summary of the trial results will be provided to parents if they have given consent for this.

#### Dissemination policy

Results will be reported in full through the National Institute for Health Research Journal series (open access), as well as through peer-reviewed journals, patient newsletters and websites.

#### Patient and public involvement

Patients and members of the public have been actively involved in the design and conduct of the CLOTHES Trial throughout.

Full details of the extent of patient and public involvement, and the impact that this has had on delivery of the trial, will be reported separately.

## Discussion

The CLOTHES Trial is an independent study that has been prioritised and commissioned by the NIHR in the United Kingdom. It will provide high-quality evidence to inform clinical decision-making on the role of silk therapeutic garments for the management of eczema in children. This pragmatic trial has been designed to reflect how silk garments might be used in normal clinical practice, and usual eczema care is allowed alongside use of the intervention.

This CLOTHES Trial has generated a significant level of public interest, and commissioners of healthcare are awaiting the trial results prior to making decisions over the future availability of silk garments through the NHS. As a result, it is likely that the results of the CLOTHES Trial will inform practice swiftly upon completion.

Our nested qualitative study is being used to inform the development of an implementation plan that is based on the views of patients, healthcare providers and commissioners, and will help to inform interpretation of the trial results and guide our dissemination plans.

We are grateful to the two companies who have contributed to the study by donating silk garments for use in this trial, and appreciate their understanding in ensuring that the trial team remain completely independent of commercial interest throughout the conduct and reporting of the study.

## Trial status

The CLOTHES Trial is ongoing. It opened to recruitment in November 2013 and aims to complete recruitment in May 2015. Data collection, analysis and write-up should be completed by the end of 2016.

## Abbreviations

ADQoL: Atopic Dermatitis Quality of Life preference-based index
AE: adverse event
ANCOVA: analysis of covariance
CHU-9D: Child Health Utility, nine dimensions
CI: chief investigator
CTU: Clinical Trials Unit
DFI: Dermatitis Family Impact Questionnaire
EASI: Eczema Area and Severity Index
EQ-5D-3 L: EuroQol Five Dimension Three Levels questionnaire
*FLG*: gene encoding filaggrin
GCP: Good Clinical Practice
GP: general practice
HTA: Health Technology Assessment
IGA: Investigator Global Assessment
ITT: intention-to-treat
NESS: Nottingham Eczema Severity Scale
NHS: National Health Service
NICE: National Institute for Health and Care Excellence
NIHR: National Institute of Health Research
PGA: Patient Global Assessment
PI: Principal Investigator at a local centre
PICS: Patient Identification Centres
POEM: Patient Oriented Eczema Measure
QALYs: Quality Adjusted Life Years
RCT: randomised controlled trial
REC: Research Ethics Committee
R&D: Research and Development
SAE: serious adverse event
SD: standard deviation
SOP: Standard Operating Procedure
TIS: Three Item Severity scale
TMG: Trial Management Group
TSC: Trial Steering Committee
